# Supplementary material for: Diagnosis of heart diseases: A fuzzy-logic-based approach
Source: PLoS One. 2024 Feb 6;19(2):e0293112. doi: 10.1371/journal.pone.0293112 (PMC10846727; doi:10.1371/journal.pone.0293112)
Supplement: Attachment — Submitted filename: PONE-D-22-26721R1-review report.pdf [file pone.0293112.s003.pdf]

Dear Editor and Author:

I have carefully reviewed the manuscript " Diagnosis of Heart Diseases: A Fuzzy-Logic-based Approach" submitted to PLOS ONE. I appreciate the authors' efforts in presenting their research on designing and creating a fuzzy logic-based expert system for the prognosis and diagnosis of heart disease. However, I have several concerns regarding the manuscript's structure, content, and clarity that need to be addressed before it can be considered for publication.

**Structure and Organization:**

The manuscript lacks a clear and well-structured organization, which makes it difficult to follow the flow of information. I suggest the authors reorganize the manuscript into sections such as Introduction, Materials and Methods, Results, Discussion, and Conclusion to provide a logical study progression.

**Methodology:**

The description of the proposed fuzzy logic-based expert system is insufficient. The authors briefly mention the system's components, such as the fuzzification module, knowledge base, inference engine, and defuzzification module, but fail to explain how these components work together comprehensively. It is crucial to provide a detailed account of the fuzzy logic techniques employed, including the membership functions, rules, and defuzzification technique, to ensure the readers can fully understand the methodology.

**Dataset and Evaluation:**

The authors mention testing the system using the Cleveland dataset and cross-checking with an in-field dataset but fail to provide specific details about these datasets. It is essential to provide information on these datasets' characteristics, size, and source. Additionally, the authors should explain the selection criteria used for the datasets and provide a thorough analysis of the evaluation results to support their claim of achieving 98.08% accuracy.

**Clarity of Figures:**

The figures in the manuscript are blurry and illegible, making it challenging to comprehend the information presented. I strongly recommend the authors improve the quality and resolution of the figures to ensure readability. Additionally, providing detailed captions and labels for each figure would enhance their clarity and help readers understand the visual information effectively.

In summary, this manuscript requires substantial revisions to improve its structure, clarify its methodology, enhance the quality of figures, and emphasize the significance of the findings. Addressing these concerns will strengthen the manuscript and ensure its suitability for publication in PLOS ONE.

I recommend the authors undertake these major revisions and resubmit the manuscript for further consideration. I would be pleased to re-evaluate the revised version once it is submitted.

Thank you for the opportunity to review this manuscript.

Sincerely,
