# Supplementary material for: Diagnosis of heart diseases: A fuzzy-logic-based approach
Source: PLoS One. 2024 Feb 6;19(2):e0293112. doi: 10.1371/journal.pone.0293112 (PMC10846727; doi:10.1371/journal.pone.0293112)
Supplement: Attachment — Submitted filename: Response to Reviewers.docx [file pone.0293112.s002.docx]

**Responses to the Comments of Reviewers**

**Reviewer 1**

**Comment 1:** Figure quality needs to be improved.

**Response 1:** Thank you for your insightful comment. We have improved the figure quality

**Comment 2:** Dataset attribute requires to represent in the paper.

**Response 2:** Thank you for your nice comment. Dataset attributes are mentioned in Section 2.1 (Data Set) of the revised paper.

**Comment 3:** The statement "Our system is based on inference rules as a knowledge base created by the close consultation of --Medical Experts." need to be justified by valid references of medical experts to select inputs.

**Response 3:** Thank you for your nice comment. We would like to mention politely that a sufficient number of inference rules are very much required to achieve acceptable accuracy from any fuzzy expert system. Our existing literature lacks in this issue which in turn makes them incapable of producing acceptable accuracy. To overcome this problem we have consulted with a cardiologist of a prestigious medical college in Bangladesh to develop an accurate and sufficient number of inference rules. The reference of the medical college is provided in the revised manuscript. As a result, it has enabled our proposed expert system to produce acceptable accuracy while conducting experiments using the Cleaveland benchmark dataset.

**Comment 4:** Justify your approach is a success amidst popular Deep Learning and Machine Learning Models. Contrast it.

**Response 4:** Thank you for your nice comment. We would like to politely mention that deep learning and machine learning are nowadays very much popular in the diagnosis of different diseases. However, in this case, we need a sufficient number of datasets (which is huge) to train the model otherwise the model will not produce an accurate result in the practical case. For the diagnosis of heart disease, the number of datasets is very limited and so fuzzy is appropriate here. Some of the researchers have proposed a hybrid of fuzzy and neural networks and achieved an improvement in the result. In this case, hybridization makes the system complex and computation intensive. So we have kept our focus on achieving accurate results through a pure fuzzy expert system.

**Reviewer 2**

**Comment 1:** Abstract need to be elaborated where proposed solution must be explained in detail.

**Response 1:** Thank you for your insightful comment. We have elaborated on the abstract where the proposed solution is explained in detail.

**Comment 2:** Change the title of paper.

**Response 2:** Thank you for your nice comment. We have changed the title in the revised manuscript.

**Comment 3:** Explain contribution of the work, try to add one more paragraph in introduction.

**Response 3:** Thank you for your nice comment. As per your comment and the comment of other reviewers, we have broken our introduction section into two sections (i) Introduction and (ii) Related work. Research gap based on existing research and our contribution to filling the gap has been mentioned in the related work section.

**Comment 4:** The flow chart steps need to be well explained more in detail.

**Response 4:** Thank you for your nice comment. Actions have been taken as per your suggestion.

**Comment 5:** Explain chest pain in detail.

**Response 5:** Thank you for your nice comment. Actions have been taken as per your suggestion.

**Comment 6:** Designing the expert system based on fuzzy logic need detail explanation.

**Response 6:** Thank you for your nice comment. Actions have been taken as per your suggestion.

**Comment 7:** Equation 1, 2 and 3 are not well explained in the text.

**Response 7:** Thank you for your nice comment. Actions have been taken as per your suggestion.

**Comment 8:** The status of heart disease is poorly explained.

**Response 8:** Thank you for your nice comment. We have explained the status of heart disease in detail in the revised manuscript.

The status of heart disease is determined based on seven input attributes/fields in the system: Chest Pain, HbA1c, HDL, LDL, Heart Rate, Age, and Blood Pressure. Depending on the values of these attributes, heart disease may or may not be present. Heart disease status is an integer value and its value lies between 0 and 10, or absence. The patient's chance of developing heart disease rises as the integer number rises. The output variable is divided into four (4) fuzzy sets (Healthy, Low Risk, Medium Risk, and High Risk). Table 4 shows these fuzzy sets with their ranges. Membership functions of **Healthy**, **LowRisk**, **MediumRisk,** and **HighRisk** fuzzy sets all are triangular. In Fig. 4(e), these membership functions are shown.

**Comment 8:** Explain table 5 in detail.

**Response 9:** Thank you for your nice comment. We have explained Table 5 in detail in the revised manuscript.

Table 5 provides a sample of a couple of inference rules. In this table, the first column shows the conditions of the inference rules (values of the attributes) and the second column shows the output (the status of the heart disease). For example, the first row of the first column mentions *If (Chest Pain is Non Anginal AND LDL is High AND HDL is Healthy AND HbA1c is Healthy AND Heart Rate is Healthy AND Blood Pressure is Normal AND Age is Mid)* as the condition of the inference rule. This condition detailed that the value of Chest Pain (attribute) is Non Anginal, the value of LDL is High, the value of HDL is Healthy, the value of HbA1c is Healthy, the value of Heart Rate is Healthy, the value of Blood Pressure is Normal, and the value of Age is Mid. The first row of the second column shows the output (status) of the heart disease for these conditions (in this case, the status is low risk). Similarly, other rows of this table are formed.

**Comment 10:** Add future directions separate section after conclusion.

**Response 10:** Thank you for your nice comment. We have added a new section “Future Directions” and updated the Conclusions section in the revised manuscript.

**Comment 11:** Try to check overall typo errors there are many errors in writing.

**Response 11:** Thank you for your nice comment. Actions have been taken as per your suggestion.

**Reviewer 3**

**Comment 1:** The abstract is not attractive. Some sentences in the abstract should be modified to make it more attractive for readers.

**Response 1:** Thank you for your insightful comment. The abstract and sentences inside the abstract have been upgraded to make it attractive.

**Comment 2:**  In Introduction section, it is difficult to understand the novelty of the presented research work. This section should be modified carefully. In addition, the main contribution should be presented correctly. Include more details in bullets.

**Response 2:** Thank you for your nice comment. As per your comment and the comment of other reviewers, we have broken our introduction section into two sections (i) Introduction and (ii) Related work. Research gap based on existing research and our contribution to filling the gap has been mentioned in the related work section.

**Comment 3:** The related work section should be included, and the healthcare monitoring systems should be discussed as follows. ‘A smart healthcare monitoring system for heart disease prediction based on ensemble deep learning and feature fusion’, ‘An intelligent healthcare monitoring framework using wearable sensors and social networking data’, ‘Automatic detection of Alzheimer’s disease progression: An efficient information fusion approach with heterogeneous ensemble classifiers’, ‘ANAF-IoMT: A Novel Architectural Framework for IoMT-Enabled Smart Healthcare System by Enhancing Security Based on RECC-VC’, Alzheimer’s disease progression detection model based on an early fusion of cost-effective multimodal data’ and ’ Type-2 fuzzy ontology–aided recommendation systems for IoT–based healthcare’. In addition, In the last lines of Literature review, highlight what overall technical gaps are observed in existing works that led to the design of proposed methodology.

**Response 3:** Thank you for your nice comment. Actions have been taken as per your suggestion.

**Comment 4:** Captions of the Figures and tables not self-explanatory. These captions should be self-explanatory, and clearly explaining the figure. Extend the description of the mentioned figures and tables to make them self-explanatory.

**Response 4:** Thank you for your nice comment.

**Comment 5:** All the fuzzy sets should be represented in one table. See the above type-2 fuzzy ontology.

**Response 5:** Thank you for your nice comment. We have shown a few sample inference rules in Table 5 of the manuscript. There are a huge number of inference rules generated in the proposed system and due to lessening the huge length of the paper, we have not shown all inference rules.

**Comment 6:** All figures are blurred, their quality should be improved.

**Response 6:** Thank you for your nice comment. We have updated the figures accordingly in the revised manuscript.

**Comment 7:** The inference rules are not correct. Authors should confirm these rules again.

**Response 7:** Thank you for your nice comment. We have worked with the UCI Cleaveland data set (the reference is also shown in the paper). The inference rules that are developed in the proposed system show 98% accuracy with respect to the classification results as shown in the data set. The variation is found in 2% of the total cases only.

**Comment 8:** The authors should discuss the above existing work in table 7.

**Response 8:** Thank you for your nice comment. Actions have been taken as per your suggestion.

**Comment 9:** The conclusion section should be revised. In addition, the future work should be included.

**Response 9:** Thank you for your nice comment. This issue is addressed in the response to Comment 10 of the second reviewer.
