# Supplementary material for: Diagnosis of heart diseases: A fuzzy-logic-based approach
Source: PLoS One. 2024 Feb 6;19(2):e0293112. doi: 10.1371/journal.pone.0293112 (PMC10846727; doi:10.1371/journal.pone.0293112)
Supplement: Attachment — Submitted filename: Response to Reviewers.docx [file pone.0293112.s004.docx]

**Responses to the Reviewers’ Comments**

We would like to thank the anonymous associate editor and reviewers for their careful reading and constructive feedback on the previous draft entitled “**Diagnosis of Heart Diseases: A Fuzzy-Logic-based Approach**”. We have carefully considered their comments in preparing our revision, which has resulted in a manuscript that is clearer, more compelling, and broader.

**Comments of Reviewer:**

....................................................................................................................................................................

**Comment #1:** *Regarding* *Structure and Organization:*

*The manuscript lacks a clear and well-structured organization, which makes it difficult to follow the flow of information. I suggest the authors reorganize the manuscript into sections such as Introduction,*

*Materials and Methods, Results, Discussion, and Conclusion to provide a logical study progression.*

**Response**: Thank you for your insightful observation. It is certainly a valuable comment to enrich the manuscript. The current manuscript is reorganized and updated as per suggestion. For details, please see the manuscript. It is highlighted by yellow color.

**Comment #2:** *Regarding Methodology:*

*The description of the proposed fuzzy logic-based expert system is insufficient. The authors briefly mention the system's components, such as the fuzzification module, knowledge base, inference engine, and defuzzification module, but fail to explain how these components work together comprehensively. It is crucial to provide a detailed account of the fuzzy logic techniques employed, including the membership functions, rules, and defuzzification technique, to ensure the readers can fully understand the methodology.*

**Response**: Thank you for your comment. I would like to mention politely that the working procedure of the proposed system is shown by the flowchart of the system (with detailed illustration) on page 7.

**Comment #3:** *Regarding Dataset and Evaluation:*

*The authors mention testing the system using the Cleveland dataset and cross-checking with an in-field*

*dataset but fail to provide specific details about these datasets. It is essential to provide information on*

*these datasets' characteristics, size, and source. Additionally, the authors should explain the selection*

*criteria used for the datasets and provide a thorough analysis of the evaluation results to support their*

*claim of achieving 98.08% accuracy.*

**Response**: Thank you for your insightful observation. For testing the proposed system, a benchmark dataset of Cleveland Clinical Foundation in the (University of California, Irvine) UCI repository has been used. It is mentioned on page 4 of the manuscript. Details of the benchmark datasets can be found in the reference # [54]. We have mentioned cross-checking with an in-field dataset. The in-field data set is an example data input of a patient from a doctor of a hospital as mentioned in reference # [53]. It is shown in Table 6 of the revised manuscript on page 23.

**Comment #4:** *Regarding Clarity of Figures:*

*The figures in the manuscript are blurry and illegible, making it challenging to comprehend the information presented. I strongly recommend the authors improve the quality and resolution of the figures to ensure readability. Additionally, providing detailed captions and labels for each figure would enhance their clarity and help readers understand the visual information effectively.*

**Response**: Thank you for your comment. I would like to mention politely that the figure is system generated and it has been enhanced as best as possible as per comment of the previous reviewers. Moreover, the quality of the given figures is tested and verified by the automated tool “PACE” as recommended by the journal.

**Comment #5:** *In summary, this manuscript requires substantial revisions to improve its structure, clarify its methodology, enhance the quality of figures, and emphasize the significance of the findings. Addressing these concerns will strengthen the manuscript and ensure its suitability for publication in PLOS ONE.*

**Response**: Thank you for your insightful observation and comments. I would like to mention politely that necessary measures have been taken as per comments where it has been possible.

I appreciate your precious comment that these would strengthen the manuscript for its publication.
